# Vascular tortuosity of the internal carotid artery is related to the *RNF213* c.14429G > A variant in moyamoya disease

**DOI:** 10.1038/s41598-019-45141-y

**Published:** 2019-06-13

**Authors:** Sungjae An, Tackeun Kim, Chang Wan Oh, Jae Seung Bang, Si Un Lee, Jaehyuk Heo

**Affiliations:** 1 Department of Neurosurgery, Seoul National University Bundang Hospital, 82, Gumi-ro 173 Beon-gil, Bundang-gu, Seongnam-si, Gyeonggi-do 13620 Republic of Korea; 2 Department of Neurosurgery, Seoul National University College of Medicine, 101 Daehak-Ro Jongno-Gu, Seoul, 03080 Republic of Korea; 3 Department of Applied Statistics, The University of Suwon, 17, Wauan-gil, Bongdam-eup, Hwaseong-si, Gyeonggi-do 18323 Republic of Korea

**Keywords:** Neurovascular disorders, Cerebrovascular disorders

## Abstract

Recent studies have implicated *RNF213* mutations in the pathogenesis of moyamoya disease (MMD). However, the underlying mechanism of disease development is not fully elucidated. Nonetheless, a possible relationship between vascular morphology and hemodynamics related with MMD has been proposed. Here, we aimed to investigate the relationship between a variant of *RNF213* and the morphology of the internal carotid artery (ICA). We enrolled bilateral MMD patients who had undergone genetic testing for *RNF213*. Patients were divided into mutant and wild-type groups. Six anatomy-specific three-dimensional coordinates were collected using magnetic-resonance angiography. From these, five vectors between two adjacent points and four angles between two adjacent vectors were calculated. The tortuosity was defined as the ratio between the actual and the linear length of the ICAs. Among 58 patients, 44 and 14 belonged to the mutant and wild-type groups, respectively. The tortuosity of ICAs was significantly lower in the mutant group (p = 0.010). The change in blood flow direction was more prominent in the wild-type group (p = 0.002). The tortuosity was significantly lower in MMD patients than normal controls (p < 0.001). Our results indicate that RNF213 could play a role in the lower tortuosity observed in patients with *RNF213* mutation.

## Introduction

Moyamoya disease (MMD) is a low-incidence disease, characterized by progressive steno-occlusive lesions at distal internal carotid arteries (ICAs)^1^. Although the etiology and pathogenesis of MMD have not been fully elucidated, *RNF213* has been identified as an important susceptibility gene related to the clinical course of MMD, and mutation of this gene possibly plays a role in the development of vasculopathies such as intracranial major artery stenosis or occlusion^2–5^. Although biotechnological advances can provide a reasonable explanation for the higher incidence of MMD in East Asia, the possible mechanism related with the characteristic anatomical sites of stenoses is still uncertain.

A few studies investigated the possible relationship between hemodynamic stress and the morphology of the ICA^6,7^. Patients with MMD were confirmed to have lower tortuosity of the intracranial-extradural ICA than normal control subjects^7^. Moreover, lower tortuosity of the ICA seemed to affect wall shear stress around the bifurcation of the ICA^6,7^. Although the temporal order between the decrease in vascular tortuosity and MMD development is not clear, vascular tortuosity influences hemodynamics, and possible relationships between hemodynamics and vascular remodeling have been proposed^8–10^.

In light of the significance of *RNF213* mutation in MMD, we aimed to investigate whether this genetic variant was associated with differences in vascular tortuosity within the MMD population using three-dimensional (3D) measurement with magnetic resonance angiography (MRA).

## Methods

### Patients

This study was approved by the institutional review board of Seoul National University Bundang Hospital (B-1902/522–112), and informed consent from patients was waived. We screened our institution’s electronic medical records and identified patients who (1) had been diagnosed with MMD based on digital subtraction angiography, (2) had undergone genetic examination for *RNF213* from November 2017 to October 2018, and (3) had undergone time-of-flight magnetic resonance angiography (TOF-MRA). Patients with unilateral MMD or a history of previous revascularization surgery were excluded because these factors could alter the flow through the ICA^11^. The same number of controls matched by age and sex were screened among those with normal MRA readings. Patients were divided into a mutant group (patients with the *RNF213* c.14429G > A mutation, regardless of its homozygous or heterozygous state) and a wild-type group. Two ICAs from each subject were analyzed as independent vessels.

### Three-dimensional measurement

The source images used in this study were derived from TOF-MRA. The original Digital Imaging and Communications in Medicine (DICOM) files were imported into a 3D image analysis tool (Slicer 4.10.1; open source software, www.slicer.org/). Six anatomy-specific 3D coordinates under the RAS axes system (Right side, Anterior side, and Superior side as corresponding for X, Y, and Z coordinates) within each intracranial ICA were defined as follows: P1, the beginning of the vertical petrous ICA at the level of the carotid canal; P2, the inflection point between the vertical petrous ICA and the horizontal petrous ICA; P3, the inflection point between the horizontal petrous ICA and the vertical cavernous ICA; P4, the inflection point between the vertical cavernous ICA and the horizontal cavernous ICA; P5, the anteriormost point of the carotid siphon; and P6, the point of emergence of the anterior choroidal artery. Each point was marked at the center of the vascular lumen in the corresponding position.

**Figure 1:**
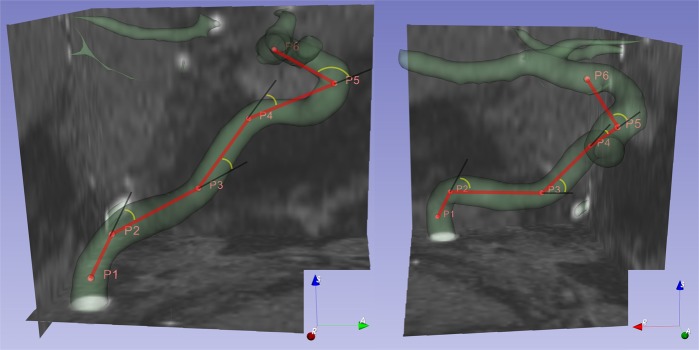
The three-dimensional reconstruction image shows each measurement under the RAS system. The image on the left shows the anatomical coordinates by right-to-left projection, while the image on the right shows the same situation by anterior-to-posterior projection. Pink points represent fiduciary points: P1, the beginning of the vertical petrous ICA at the level of the carotid canal; P2, the inflection point between the vertical petrous ICA and the horizontal petrous ICA; P3, the inflection point between the horizontal petrous ICA and the vertical cavernous ICA; P4, the inflection point between the vertical cavernous ICA and the horizontal cavernous ICA; P5, the anteriormost point of the carotid siphon; P6, the point of emergence of the anterior choroidal artery. Thick red lines are correlated with the vectors between two adjacent points. Yellow arcs represent the change in the flow directions.

Using 3D coordinates, five vectors and angles were defined. The first vector was calculated as $\overrightarrow{{\rm{V}}12}=\overrightarrow{{\rm{P}}2-{\rm{P}}1}$, which indicated the vertical petrous segment. The following $\overrightarrow{{\rm{V}}23}$, $\overrightarrow{{\rm{V}}34}$, $\overrightarrow{{\rm{V}}45}$, and $\overrightarrow{{\rm{V}}56}$ vectors were calculated in the same manner, indicating horizontal petrous, vertical cavernous, horizontal cavernous, and intracranial segments, respectively. The first angle was defined as $\angle 123={\cos }^{-1}(\frac{\overrightarrow{{\rm{V}}12}\cdot \overrightarrow{{\rm{V}}23}}{|\overrightarrow{{\rm{V}}12}|\times |\overrightarrow{{\rm{V}}23}|})$, which indicated the inter-petrous (between the vertical petrous ICA and the horizontal petrous ICA) angle. The ∠234, ∠345, and ∠456 angles were calculated in the same manner, indicating petro-cavernous, inter-cavernous, and siphon angles, respectively. Figure 1 summarizes each measurement. The actual length (ALEN) of the measured ICA was defined as $|\overrightarrow{{\rm{V}}12}|+|\overrightarrow{{\rm{V}}23}|+|\overrightarrow{{\rm{V}}34}|+|\overrightarrow{{\rm{V}}45}|+|\overrightarrow{{\rm{V}}56}|$, and the linear length (LLEN) was as defined as $|\overrightarrow{{\rm{P}}6-{\rm{P}}1}|$. The tortuosity was defined as $(\frac{{\rm{ALEN}}-{\rm{LLEN}}}{{\rm{LLEN}}})\times 100$.

As a person has two ICAs, each measured value from the right and left sides was averaged as an individual value. The more advanced Suzuki grade between the right and left side was used as the representative grade.

### Statistical analysis

Statistical analyses were performed using R (version 3.5.1, open source software, www.r-project.org). All categorical variables (sex and Suzuki grade) are presented as a number (population percentage) and were analyzed by Fisher’s exact test. All continuous variables are presented as medians (interquartile range; IQR) because some variables showed non-normal distribution by the Kolmogorov–Smirnov test. Those variables were analyzed by the Kruskal–Wallis test and Wilcoxon’s rank-sum test. Bonferroni’s correction was also applied for significant variables in the Kruskal–Wallis test. All length variables were measured in millimeters, whereas angle variables were in degrees. A p-value of < 0.05 was considered statistically significant.

## Results

### Patient characteristics

In one year (from November 2017 to October 2018), a total of 208 patients had undergone genetic tests for the *RNF213* gene (c.14429G > A mutation). Among them, 70 and 45 patients were excluded due to the lack of cerebral angiography and MRA data, respectively. Among the remaining 93 patients, 27 patients with unilateral MMD or those who underwent any prior revascularization surgery were excluded. The resultant 66 patients were divided into two groups. The mutant group comprised 52 (78.8%) patients with heterozygotic (GA) mutation.; the wild-type (without mutation) group comprised 14 (21.2%) patients. There was no patient with homozygotic mutation (AA). All the patients included in this study were Koreans. When medical records were reviewed, there was a single MMD patient (1.5%) with neurofibromatosis type 1 in mutant group. Eight patients with Suzuki grade 1 or 5 were excluded in the main analysis, because all wild-type group patients had Suzuki grade of either 2, 3, or 4.

The median age of the 58 enrolled patients was 35.5 (28.0–47.0) years. The distribution of age was not statistically different between the mutant group (34.0 [26.8–48.3]) and the wild-type group (42.5 [35.0–44.5]; p = 0.210). Among the patients, 35 (60.3%) were women. The proportion of female patients in the mutant group (61.4% [27/44]) was not significantly different from that in the wild-type group (57.1% [8/14]; p = 1.000). More patients with advanced Suzuki grade were in the mutant group; however, this was not a statistically significant finding (p = 0.056). The age and sex ratios were exactly the same between MMD patients and control subjects.

### Tortuosity analysis between mutant and wild-type *RNF213*

**Figure 2:**
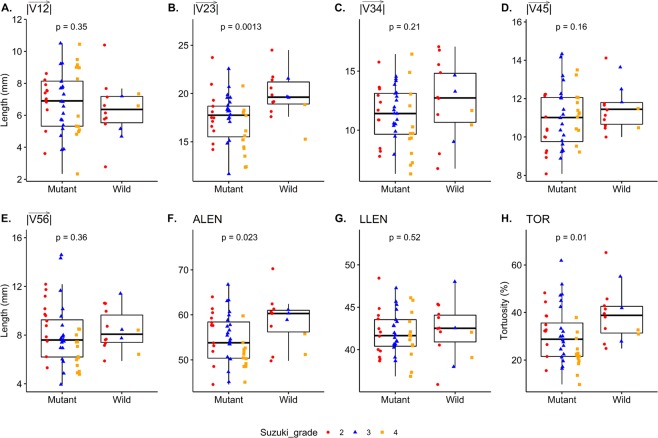
Bar plots with jitters show the distribution of the lengths and corresponding p-values. The shape and color of each jitter represents the Suzuki grade. $|\overrightarrow{{\rm{V}}12}|$, vertical petrous segment; $|\overrightarrow{{\rm{V}}23}|$, horizontal petrous segment; $|\overrightarrow{{\rm{V}}34}|$, vertical cavernous segment; $|\overrightarrow{{\rm{V}}45}|$, horizontal cavernous segment; $|\overrightarrow{{\rm{V}}56}|$, intracranial segments; ALEN, actual length; LLEN, linear length; TOR, tortuosity.

**Figure 3:**
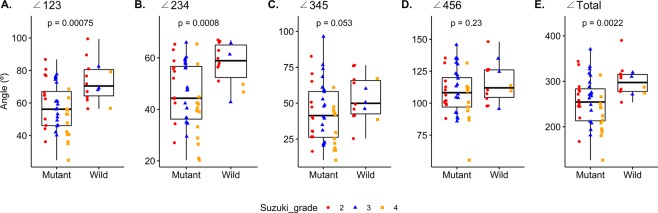
Bar plots with jitters show the distribution of the angles and the corresponding p-values. The shape and color of each jitter represents the Suzuki grade. $\angle 123,\,\,$inter-petrous angle; ∠234, petro-cavernous angle; ∠345, inter-cavernous angle; ∠456, siphon angle.

As to segmental lengths, $|\overrightarrow{{\rm{V}}23}|$ was significantly shorter (p = 0.001) in the mutant than in the wild-type group. Other segmental lengths were similar. As to the sum of segmental lengths, ALEN was significantly shorter in the mutant group (53.80 [50.38–58.41]) than in the wild-type group (60.31 [56.20–61.00], p = 0.023). Considering the similar length of LLEN between the two groups (p = 0.518), the tortuosity of ICAs was calculated to be significantly lower in the mutant group (p = 0.010) (Fig. 2). Measured angles, ∠123 and ∠234, showed that the angle change of the ICA was significantly lower in the mutant group. Other angles, ∠345 and ∠456, were similar in each comparison. The total change in blood flow direction was more prominent in the ICAs of wild-type group patients (p = 0.002, Fig. 3).

### Tortuosity analysis among MMD patients and controls

Table 1 summarizes the measured parameters and statistical analyses. The sum of all segmental lengths was the longest among control subjects (64.07 [58.43–67.06]; p < 0.001), while the linear length between P1 and P6 was similar among patient groups (p = 0.818). Control subjects showed the highest tortuosity (46.52 [43.04–59.21]), followed by wild-type MMD patients (38.81 [31.36–42.57]) and mutant MMD patients (28.72 [21.51–35.58]; p < 0.001). Bonferroni’s correction showed pair-wise statistical differences as well. Similarly, the total change in blood flow direction was the highest among control subjects (313.0 [279.8–352.1]) and the lowest among mutant MMD patients (254.5 [213.2–283.5], p < 0.001). In pair-wise comparisons, mutant MMD patients had a greater angle change than control individuals (p < 0.001) and wild-type MMD patients (p = 0.005). However, the total change in angle was similar between wild-type MMD patients and control individuals (p = 0.654). In short, although the tortuosity of wild-type MMD patients was lower than that of control subjects, the difference was more pronounced in the mutant MMD group.

**Table 1:** Data summary.

		Group			*p*-values			
		Mutant (n = 44)	Wild (n = 14)	Control (n = 58)	Overall	Mutant/Wild	Mutant/Control	Wild/Control
Age		34.0 (26.8–48.3)	42.5 (35.0–44.5)	35.5 (28.0–47.0)	0.452			
Sex (Female)		27 (61.4%)	8 (57.1%)	35 (60.3%)	1.000			
Suzuki grade	2	12 (27.3%)	9 (64.3%)			0.056		
	3	19 (43.2%)	3 (21.4%)					
	4	13 (29.5%)	2 (14.3%)					
$|\overrightarrow{V12}|$ (mm)		6.90 (5.32–8.14)	6.37 (5.53–7.18)	7.67 (6.07–8.78)	0.088			
$|\overrightarrow{V23}|$ (mm)		17.75 (15.51–18.69)	19.62 (18.91–21.20)	19.24 (18.23–20.45)	<0.001	0.003	<0.001	1.000
$|\overrightarrow{V34}|$ (mm)		11.41 (9.66–13.12)	12.73 (10.67–14.81)	13.41 (11.71–15.73)	<0.001	0.627	<0.001	0.888
$|\overrightarrow{V45}|$ (mm)		11.01 (9.76–12.06)	11.44 (10.66–11.80)	11.87 (10.66–13.13)	0.024	0.498	0.024	1.000
$|\overrightarrow{V56}|$ (mm)		7.60 (6.20–9.26)	8.08 (7.40–9.65)	10.78 (9.72–11.72)	<0.001	1.000	<0.001	<0.001
ALEN (mm)		53.80 (50.38–58.41)	60.31 (56.20–61.00)	64.07 (58.43–67.06)	<0.001	0.064	<0.001	0.036
LLEN (mm)		41.65 (40.40–43.54)	42.51 (40.91–44.07)	41.65 (40.38–43.87)	0.818			
Tortuosity (%)		28.72 (21.51–35.58)	38.81 (31.36–42.57)	46.52 (43.04–59.21)	<0.001	0.027	<0.001	0.009
∠123 (°)		56.18 (46.11–67.10)	70.45 (64.40–80.54)	72.09 (61.33–82.82)	<0.001	0.001	<0.001	1.000
∠234 (°)		44.32 (36.21–56.67)	58.89 (52.34–65.00)	60.51 (54.35–66.53)	<0.001	0.001	<0.001	1.000
∠345 (°)		41.37 (26.26–58.13)	49.91 (42.56–65.79)	61.08 (42.66–68.82)	<0.001	0.157	<0.001	1.000
∠456 (°)		108.18 (96.99–119.95)	111.99 (104.55–126.06)	126.46 (118.83–136.79)	<0.001	0.710	<0.001	0.090
Total angle (°)		254.49 (213.20–283.53)	297.38 (277.78–315.11)	313.00 (279.75–352.10)	<0.001	0.005	<0.001	0.654

Suzuki grade, presented as number (percent), was analyzed by Fisher’s exact test between the mutant and wild-type group. The proportion of female patients is presented as number (percent), and it was analyzed by Fisher’s exact test among the three groups. Other variables are presented as median (interquartile range) and were analyzed by the Kruskal–Wallis test with Bonferroni’s corrections for statistical difference. V12, vertical petrous segment; V23, horizontal petrous segment; V34, vertical cavernous segment; V45, horizontal cavernous segment; V56, intracranial segment; ALEN, actual length; LLEN, linear length; ∠123, inter-petrous angle; ∠234, petro-cavernous angle; ∠345, inter-cavernous angle; ∠456 siphon angle.

### Tortuosity and Suzuki grade

**Figure 4:**
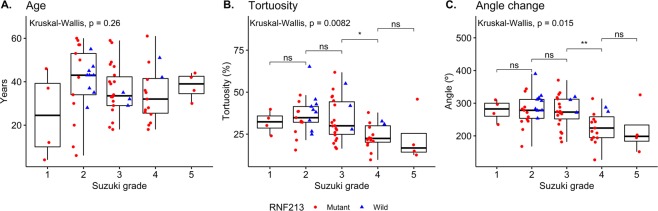
Bar plots with jitters show the distribution of the tortuosity and the angles with corresponding p-values according to Suzuki grade. The shape and color of each jitter represents the mutation status. ns, not significant; *p < 0.05; **p < 0.01.

Supplementary analysis among all patients, including those with Suzuki grades 1 and 5, was performed. In this analysis, 52 and 14 patients were included in the mutant and wild-type groups, respectively. The median tortuosity of ICAs was significantly lower, 28.72 (21.39–35.58), in the mutant group, whereas it was 38.81 (31.36–42.57, p = 0.008) in the wild-type group. Total angle change of flow direction was also significantly lower in the mutant group (254.5 [210.7–287.8]) than in the wild-type group (297.4 [277.8–315.1]; p = 0.002). As for Suzuki grade, the age distribution was similar among grade groups. The overall tortuosity and change in angle tended to decrease with a higher grade, although the differences between adjacent grades were not statistically significant except between grade 3 and 4 (Fig. 4).

## Discussion

The protein RNF213, encoded by the *RNF213* gene, is known to be associated with angiogenesis^12^. RNF213 deficiency in the endothelium leads to the overexpression of several matrix metalloproteinase genes. This deficiency has deleterious effects on endothelial cells in the maintenance of vascular structure^13^. Sonobe *et al*. revealed that mice lacking RNF213 showed suppressed vascular remodeling after common carotid artery ligation^14^. The ischemic insult caused increased vascular expression of matrix metalloproteinase-9 and thinning of the vascular wall distal to the ligation. Similarly, *RNF213* knockout mice showed enhanced angiogenesis following hindlimb ischemia. This enhancement in angiogenesis corresponds to the abnormal cerebral vascular network in the chronic ischemic state. However, after cerebral ischemia, no significant difference in brain angiogenesis was found compared with the wild-type^15^.

However, vasculopathies that are strongly related to genetic mutation tend to involve multisystemic vessels. Since MMD inarguably involves a specific part of the vessel, some efforts have been made to study the *RNF213* c.14429G > A mutation and its relationship with intracranial vascular morphology^16,17^. However, the link between the vasculopathies and the specific location of the MMD culprit, i.e., the distal ICA, remains unsolved. Computational fluid dynamics demonstrated that lower ICA tortuosity results in higher wall shear stress, especially at the terminal ICA^6,7^. Thus, the hypothesis derived here was that the *RNF213* c.14429G > A mutation is related to distal ICA stenosis/occlusion of MMD through the intracranial ICA phenotype involving tortuosity.

We showed the morphological differences of the ICA according to the mutation status of *RNF213* c.14429G > A. Although the most accurate method for calculating the tortuosity of vessels is the extraction of centerlines from 3D-rendered vascular models produced by manual segmentation of a source image, its analysis requires excessive time and manpower^7^. Thus, in this study, we adopted a method for defining six landmark points and calculating the straight lines connecting the adjacent points, thereby approximating the trajectory of the actual vessels. By plotting 3D coordinates using the patients’ MRA, we could obtain segmental length, angle, and tortuosity of the vessel close to real values, such that an acceptable data comparison could be made. Through the analysis, we could show the lower tortuosity and flow angle change of the ICA in MMD patients with mutated *RNF213* c.14429G > A compared with those in wild-type MMD patients. Considering the relationship among tortuosity, *RNF213* mutation, and Suzuki grade, it could be inferred that patients with *RNF213* mutation would be diagnosed with MMD in more advanced status, and that differences in one of the phenotypes would result in vascular tortuosity.

Numerous studies have been carried out to investigate the pathogenesis of MMD, and complex regulation of angiogenesis or vasculopathy have been revealed, with some contradictory results between studies^18^. If gross vascular morphological characteristics related with the *RNF213* c.14429G > A mutation can be demonstrated, various angiogenic or vasculopathy properties of MMD can be understood as a gross phenomenon. Our study successfully revealed the relationship between the *RNF213* c.14429G > A mutation and greater ICA tortuosity.

A crucial limitation of this study was related to the design of the study. Since we performed this investigation as a retrospective case-control study at a certain time, we could only show correlations between the morphological differences of the ICA and the *RNF213* c.14429G > A mutation and were unable to prove causality. Serial tortuosity of the ICA and cerebral perfusion need to be measured throughout the disease course for validating the causality. Another limitation was the modest sample size. Although over 200 subjects underwent genetic testing for the *RNF213* c.14429G > A mutation, almost 68% were excluded to secure a homogenous study population (bilateral MMD, availability of cerebral angiography and MRA). As only 14 subjects were allocated to the wild-type group with Suzuki grade 2–4, additional eight subjects were also excluded from mutant group who presented as Suzuki grade 1 or 5. The distributions of sex and age were statistically similar between the two groups. Although the distribution of Suzuki grade showed statistical homogeneity, the p-value was marginal. Thus, we performed additional analysis among the tortuosity, angle change, and Suzuki grade which showed that ICA tortuosity tended lower in earlier Suzuki stage patients.

Another potential weakness of this study was related to the simplified methodology of measurement. A previous tortuosity measurement was made by actual measurement after 3D centerline extraction following segmentation and rendering^7^. Although 3D segmentation and centerline extraction is the most accurate method to measure vascular morphology, it requires considerable effort. Our methodology used a simplified method approximating real measurement and required the measurement of only six fiducial points, which could be easily obtained using an open source software. Although the accuracy might be slightly decreased, the range of measured tortuosity seemed to be acceptable compared with that of a previous report, considering the longer segment investigated (to the point of emergence of the anterior choroidal artery) than that in the earlier report (to the carotid siphon)^7^.

Together, our results indicate that RNF213 might be involved in the shaping of the ICA, with *RNF213* deficiency resulting in terminal ICA stenosis/occlusion through focal high wall shear stress from the lower tortuosity of conduits^6,7^. Further serial observational studies using a larger disease cohort are warranted for further clarification of the causal relationship between the morphology of the ICA and MMD.

## Data Availability

The data are not available for public access because of patient privacy concerns but are available from the corresponding author on reasonable request approved by the institutional review boards of Seoul National University Bundang Hospital.
