# Author Correction: Vascular tortuosity of the internal carotid artery is related to the *RNF213* c.14429G > A variant in moyamoya disease

**DOI:** 10.1038/s41598-020-60645-8

**Published:** 2020-02-28

**Authors:** Sungjae An, Tackeun Kim, Chang Wan Oh, Jae Seung Bang, Si Un Lee, Jaehyuk Heo

**Affiliations:** 1 Department of Neurosurgery, Seoul National University Bundang Hospital, 82, Gumi-ro 173 Beon-gil, Bundang-gu, Seongnam-si, Gyeonggi-do 13620 Republic of Korea; 2 Department of Neurosurgery, Seoul National University College of Medicine, 101 Daehak-Ro Jongno-Gu, Seoul, 03080 Republic of Korea; 3 Department of Applied Statistics, The University of Suwon, 17, Wauan-gil, Bongdam-eup, Hwaseong-si, Gyeonggi-do 18323 Republic of Korea

Correction to: *Scientific Reports* 10.1038/s41598-019-45141-y, published online 13 June 2019

This Article contains errors in Reference 6 which was incorrectly given as:

Sudhir, B. In International Conference on Intelligent Informatics and Biomedical Sciences (ICIIBMS). 72–77 (IEEE) (2018)

The correct reference is listed below as ref. 1:
